# Mouthguard use and awareness among athletes in Turkey: a cross-sectional study with multivariable analysis

**DOI:** 10.1186/s13102-025-01377-y

**Published:** 2025-11-17

**Authors:** Başak Topdaği, Gizem Yazdan Özen, Ali Kağan Özen

**Affiliations:** 1 Faculty of Dentistry, Department of Prosthetic Density, Health Sciences University Hamidiye Campus, Selimiye, Tıbbiye Cd, Üsküdar/İstanbul, 34668 Türkiye; 2 Faculty of Dentistry, Department of Orthodontics, Kafkas University, Kars, Türkiye Turkey; 3 Faculty of Dentistry, Department of Orthodontics, Kafkas University, Kars, Türkiye Turkey

**Keywords:** Mouthguard, Sport, Injury, Dental trauma, Mouth trauma

## Abstract

**Objectives:**

This prospective cross-sectional study aimed to examine mouthguard use and awareness during sporting activities.

**Methods:**

A 30-question demographic survey, developed by researchers, was administered to 411 athletes aged 18 and older who are actively involved in various sports across Turkey. The surveys included demographic information, mouthguard use, knowledge, and awareness. Age mean of mouthguard users was 22.97 ± 3.19, and age mean of non-users were 22.80 ± 4.12. Among the women, 11.6% sometimes and 8.0% always used mouthguard; among the men, 9.1% sometimes and 7.0% always used mouthguard.

**Results:**

Mouthguard use did not differ significantly based on gender, tooth brushing frequency, duration of sports activity, license status, or license type (*p* > 0.05). However, mouthguard use was statistically significantly higher among those who visited the dentist more frequently, those who practiced combat sports and boxing, those who exercised more frequently on weekdays, those who had general knowledge about mouthguards, and those who were aware of their benefits (*p* < 0.05). Mouthguard usage was significantly correlated with dentistry visit frequency (*r* = 0.214; *p* < 0.01), sport frequency (*r* = 0.244; *p* < 0.01), license (*r* = 0.111; *p* < 0.05), mouthguard general knowledge (*r* = 0.364; *p* < 0.01) and mouthguard benefits knowledge (*r* = 0.454; *p* < 0.01). Effects of sport frequency (B = 0.496; *p* < 0.01) and mouthguard benefit knowledge (B = 2.240; *p* < 0.01) on mouthguard usage (mouthguard benefit knowledge, OR = 9.396) were statistically significant.

**Conclusion:**

Although the majority of participants participated in sports involving collisions and those with a high risk of oral trauma, mouthguard use and awareness were quite low. Public health and clinical dentistry fields must work together to increase mouthguard knowledge.

**Supplementary Information:**

The online version contains supplementary material available at 10.1186/s13102-025-01377-y.

## Introduction

Mouthguards are devices used particularly in sports training and competitions to prevent orofacial tissue damage from trauma or similar causes [1]. In addition to tissue damage, orofacial trauma can lead to facial intrusion, avulsion, soft tissue injuries, facial bone trauma, and tooth fractures [2]. A properly prepared and used mouthguard minimizes damage to hard and soft tissues and provides a significant quality of life for the patient, eliminating the need for treatment [3]. Mouthguards produced for this purpose protect the oral and facial health of individuals, while interactive versions can be controlled by phones, computers, or wheelchairs [4]. The common goal of all these products can be summarized as maximizing oral and facial health while simultaneously ensuring functionality. Furthermore, instrumented mouthguards have been shown to prevent not only dental and facial health but also head and brain trauma [5]. Although there are many new types and 3D alternatives today [6], the most common types can be classified as Stock Type [7], Boil-and-Bite [8], Custom [9] and Instrumented [10]. Types of mouthguards were shown in the Table 1.

**Table 1:** Types of mouthguards

Type	Properties
Stock Type	Although stock mouthguards are prefabricated and ready to use right out of the box, they might not fit perfectly.
Boil-and-Bite	Generally constructed from medical-grade silicon or plastic.
Custom	Customized according to user requirements.
Instrumented	Modified according to user requirements.

Although mouthguards play an effective role in preventing all head traumas and facial injuries, they primarily focus on oral and dental protection. In the dental field, increased tooth mobility due to injuries, particularly in athletes, is increasing the need for orthodontic applications, and mouthguards play a significant role in preventing this [11]. Therefore, both coaches and dentists should guide their sports patients to recommend mouthguard use during sports competitions or training [12]. Meta-analyses on the effect of mouthguard use in sports activities have reported that the risk of injury without mouthguard use is between 1.6 and 1.9 [13]. This applies to facial tissues and bones, as well as teeth [14]. Therefore, it is possible to say that mouthguards play an important role in dental health.

Despite the importance of mouthguards for dental and oral health, their use and general knowledge of them in situations where oral trauma or injury may occur, particularly in sports, are quite limited [15, 16]. Furthermore, studies providing quantitative information on mouthguard use by athletes are limited, resulting in a lack of literature in this regard. This study aimed to examine mouthguard use and awareness during athletic activities.

## Methods

### Research model

The study was designed using a mixed model, including cross-sectional and retrospective descriptive and correlational screening models. In retrospective descriptive model, research issue is described from retrospectively collected data. The descriptive screening model first identified athletes’ mouthguard use and knowledge levels. The correlational screening model was then used to analyze which variables supported and significantly influenced mouthguard use.

### Participants

The research population consists of individuals aged 18 and over who are actively involved in various sports branches across Turkey. The sample was created through voluntary participation on online platforms. The survey form, prepared using Google Forms, was distributed to participants via social media, email, and the digital networks of sports communities. The study is descriptive and cross-sectional in nature. The analysis, conducted using the G*Power 3.1 program, was based on an effect size of 0.30, α = 0.05, and a power level of 80%. A minimum of 88 participants was calculated. To increase representativeness, the sample size was targeted at 150–200. This number was exceeded in the study, and 411 participants were included. The inclusion criteria for the study were as follows:

- Participating in amateur or professional sports.
- Being over 18.
- Possessing basic literacy skills.
- Voluntarily participating in research.

Since the sample was recruited entirely through online platforms, the potential risk of selection bias was avoided by participant consent form at the beginning of online surveys.

### Data collection tools

Data were collected using a structured survey form developed by the researcher. The survey’s content validity was assessed by field experts. The form consists of 30 questions and includes five main topics:

- Demographic information (age, gender, sports history).
- Oral and dental health habits (brushing, frequency of dental visits).
- Trauma history (facial/dental injury experience).
- Mouthguard awareness (use status, knowledge level).
- Usage experience and habits (frequency of use, preferred type, cleaning).

The first page of the survey includes an informed consent box, emphasizing the purpose of the study, confidentiality principles, and voluntary participation. Individuals who do not consent will not be directed to the survey.

Surveys were administered to participants via an online form developed by the researcher. Participants were asked to complete a consent form and declare their willingness to participate in the survey. Data collection was conducted in accordance with the criteria of the Declaration of Helsinki.

The dimension measuring knowledge about mouthguards and their general usage characteristics was defined as “general knowledge.” The benefits of mouthguard use to the user were defined as “mouthguard benefit knowledge.”

Since the questions were demographic, that is, in question form, and did not include a Likert-type evaluation, the Cronbach Alpha value was not considered as internal consistency, but was evaluated based on expert consistency.

### Procedure

Data collection will be conducted entirely online; no face-to-face interviews, phone calls, or emails were conducted. Participants accessed and responded to the form using Google Forms on their own devices. The average completion time is 6–8 min. No personal identifying information is requested; data is recorded completely anonymously.

This study is based solely on the administration of a survey; there are no physical, biological, or medical interventions, clinical tests, or laboratory procedures. Participants will not be subjected to any research-specific procedures. All procedures performed within the scope of the research are specific to the study and do not involve routine clinical practice.

###  Ethical approval

Ethics committee approval for the study was obtained from the Kafkas University Non-Interventional Clinical Research Ethics Committee, dated May 28, 2025, with decision number KA-TFEK 2025-05/07. The research was planned in accordance with the Declaration of Helsinki. Following ethics committee approval, the survey was launched. The first page of the survey clearly explained the principles of voluntary participation, data confidentiality, and the anonymity of participants. Participants were able to proceed with the survey after checking the informed consent box.

### Statistical methods

Nominal and ordinal data were described using frequencies, and measured data were described using mean and standard deviation. Chi-square and likelihood tests were used for variance analyses. In correlation analyses, mouthguard use was used as a dummy variable, and Spearman’s rho correlation was performed. Due to linearization deviations [17, 18], Binary Logistic Regression analysis was used for relational screening analysis. All analyses were performed using SPSS 25.0 for Windows at a 95% confidence interval and a significance level of 0.05.

## Results

Among the women, 11.6% sometimes and 8.0% always used mouthguard; among the men, 9.1% sometimes and 7.0% always used mouthguard. There was no statistically significant difference between the levels of mouthguard use according to gender (*p* > 0.05). Among the participants who never brushed their teeth, 33.3% sometimes, 16.7% of those who brushed very rarely always, 9.7% of those who brushed rarely always, 6.8% of those who brushed once a day and 6.6% of those who brushed twice a day stated that they used mouthguard regularly. There was no statistically significant difference between the mouthguard use patterns according to the frequency of tooth brushing (*p* > 0.05). 10.0% of those who never went to the dentist, 4.7% of those who went to the dentist because of a complaint, 6.5% of those who went to the dentist once a year, 13.5% of those who went to the dentist twice a year, and 20.8% of those who went to the dentist four times a year stated that they used mouthguards regularly. The differences in mouthguard use according to the annual frequency of going to the dentist were not statistically significant (*p* > 0.05). 35.3% of boxers, 22.7% of combat sports, 1.4% of football players, 8.3% of swimmers, 10.0% of tennis players, and 5.3% of those who did more than one sport stated that they used mouthguards regularly. Mouthguard use showed a statistically significant difference according to the type of sport (*p* < 0.05). Mouthguard use rates did not show a statistically significant difference according to the year of sports (*p* > 0.05). According to the frequency of doing sports, 2.8% of those who do sports 1–2 times a week, 8.5% of those who do sports 3–4 times a week, 21.8% of those who do sports 5–6 times a week, and 10.3% of those who do sports regularly stated that they use mouthguards regularly, and these differences were statistically significant (*p* < 0.05). The differences between the mouthguard usage levels of licensed and unlicensed athletes were not statistically significant (*p* > 0.05). No statistically significant difference was observed between the mouthguard usage levels according to the license type (*p* > 0.05). 14.0% of those who had knowledge about mouthguards and 16.0% of those who knew about their benefits stated that they use mouthguards regularly, and the differences between the groups were statistically significant (*p* < 0.05) (Table 2).

**Table 2:** Baseline characteristics of mouthguard users and difference analysis results

		Mouthguard usage						*p* value
		No		Sometimes		Always		
		Frequency (*n*)	Percent (%)	Frequency (*n*)	Percent (%)	Frequency (*n*)	Percent (%)	
Gender	Female	90	80.4	13	11.6	9	8.0	0.680^a^
	Male	250	83.9	27	9.1	21	7.0	
Tooth brush frequency	None	2	66.7	1	33.3	0	0.0	0.674^b^
	Very rare	15	83.3	0	0.0	3	16.7	
	Rare	26	83.9	2	6.5	3	9.7	
	Once in a day	123	84.2	13	8.9	10	6.8	
	Twice in a day	174	82.1	24	11.3	14	6.6	
Dentistry yearly visit frequency	Never	26	86.7	1	3.3	3	10.0	0.000^b^
	On complaint	229	88.8	17	6.6	12	4.7	
	Once	35	76.1	8	17.4	3	6.5	
	Twice	33	63.5	12	23.1	7	13.5	
	Four times	17	70.8	2	8.3	5	20.8	
Sport branch	Athletics	10	100.0	0	0.0	0	0.0	0.009^b^
	Badminton	6	100.0	0	0.0	0	0.0	
	Basketball	13	100.0	0	0.0	0	0.0	
	Boxing	14	41.2	8	23.5	12	35.3	
	Fight sports	10	45.5	7	31.8	5	22.7	
	Football	66	93.0	4	5.6	1	1.4	
	Volleyball	38	88.4	5	11.6	0	0.0	
	Swimming	11	91.7	0	0.0	1	8.3	
	Tennis	9	90.0	0	0.0	1	10.0	
	Multi	162	86.2	16	8.5	10	5.3	
Sport year	Under 1 year	46	86.8	4	7.5	3	5.7	0.620^b^
	1–2 years	29	85.3	4	11.8	1	2.9	
	3–4 years	57	78.1	7	9.6	9	12.3	
	> 5 years	208	83.2	25	10.0	17	6.8	
Sport weekly frequency	1–2 times	166	91.7	10	5.5	5	2.8	0.000^b^
	3–4 times	88	75.2	19	16.2	10	8.5	
	5–6 times	36	65.5	7	12.7	12	21.8	
	All days	22	75.9	4	13.8	3	10.3	
License	No	151	87.8	14	8.1	7	4.1	0.052^a^
	Yes	189	79.4	26	10.9	23	9.7	
License type	Amateur	163	80.3	21	10.3	19	9.4	0.208^a^
	Professional	48	70.6	9	13.2	11	16.2	
Mouthguard related profile								
Mouthguard general knowledge	No	225	94.5	7	2.9	6	2.5	0.000^a^
	Yes	115	66.9	33	19.2	24	14.0	
Mouthguard benefits knowledge	No	254	95.5	5	1.9	7	2.6	0.000^a^
	Yes	86	59.7	35	24.3	23	16.0	

^a^Chi-Square

^b^Likelihood Ratio

**Fig. 1:**
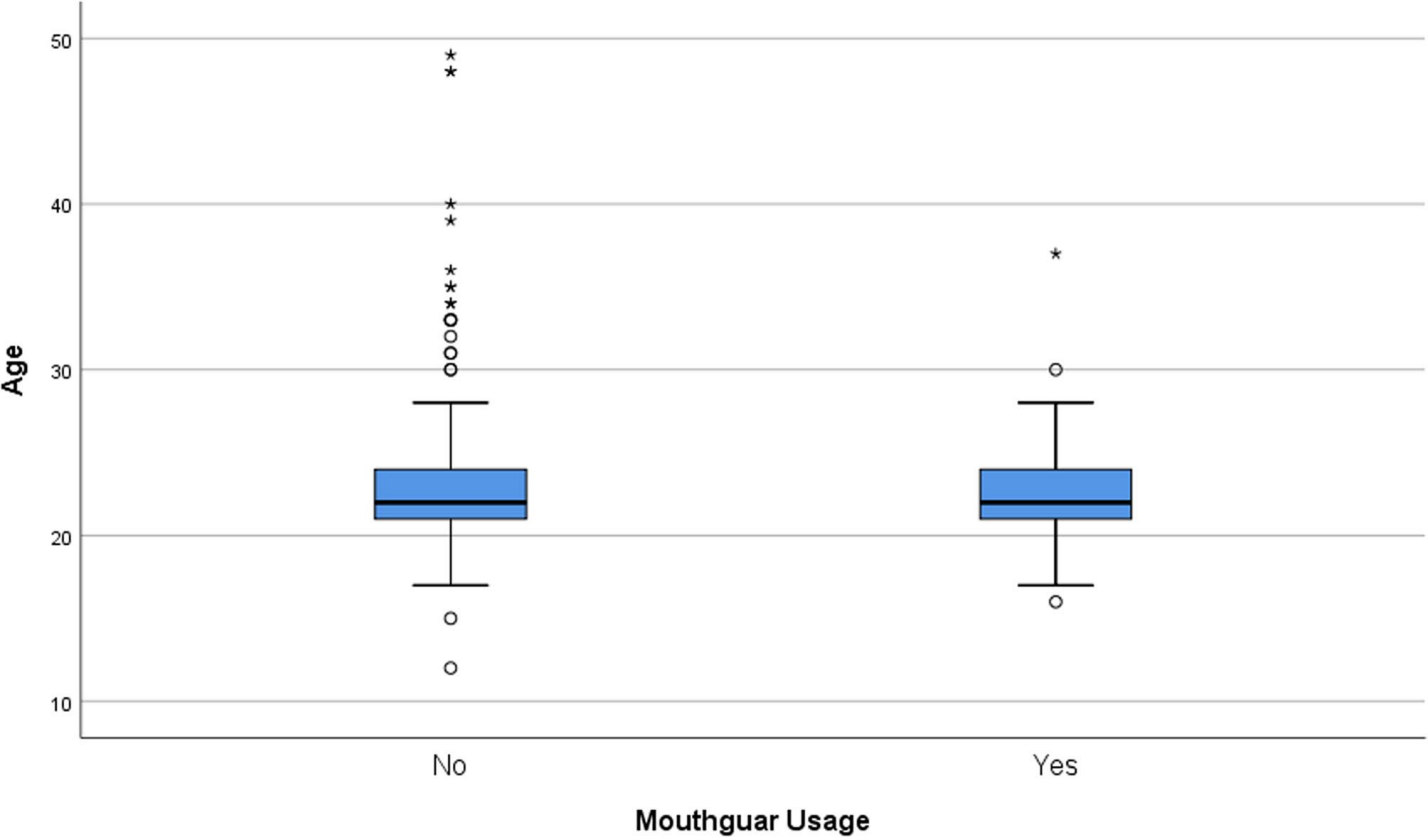
Age means and ranges of mouthguard users and not users

**Fig. 2:**
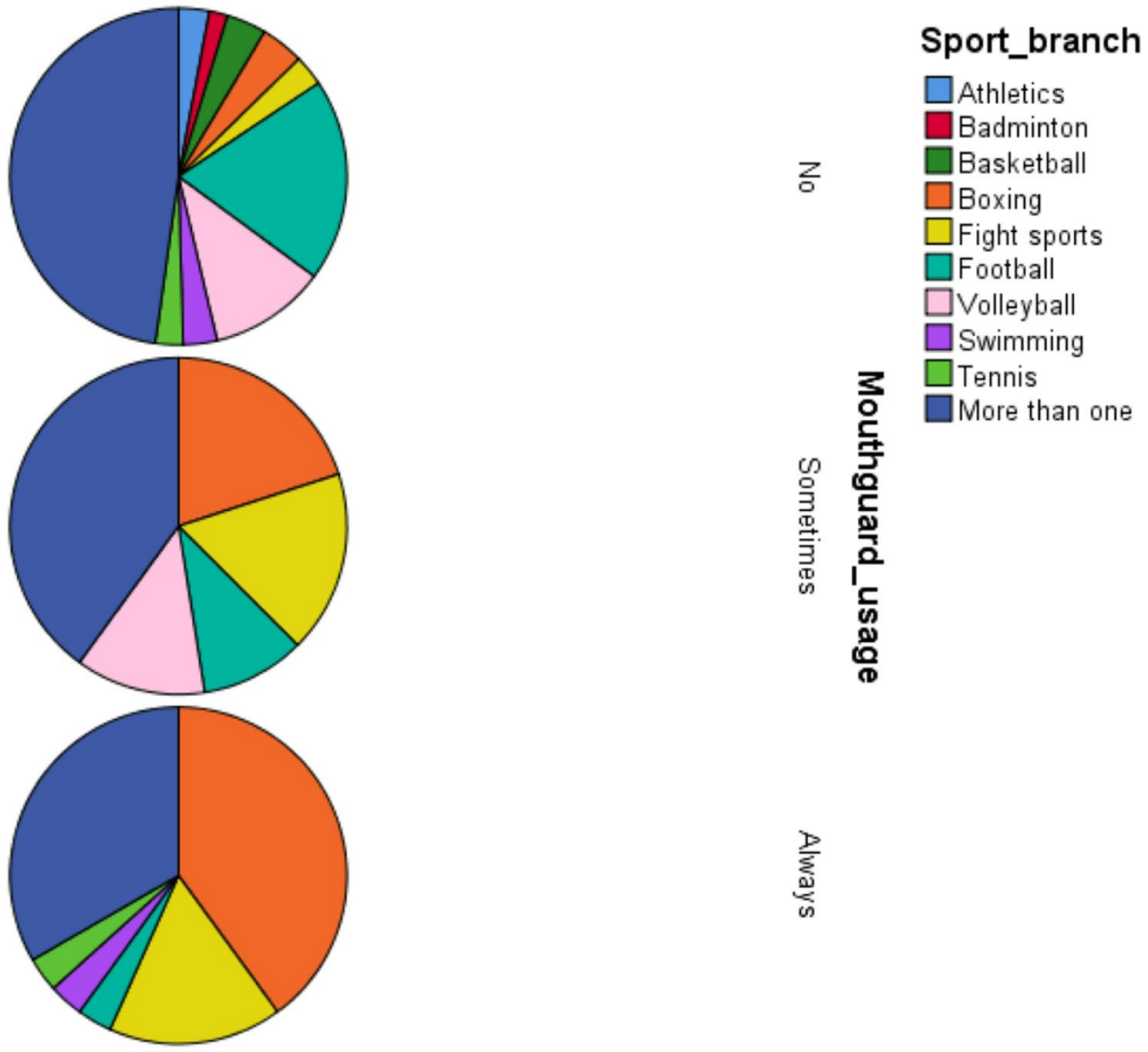
Mouthguard usage according to branches

Age mean of mouthguard users was 22.97 ± 3.19, and age mean of non-users were 22.80 ± 4.12 with statistically insignificant difference (*p* > 0.05) (Fig. 1). Mouthguard usage according to branches was also shown in Fig. 2.

Mouthguard usage was significantly correlated with dentistry visit frequency (r=0.214; p<0.01), sport frequency (r=0.244; p<0.01), license (r=0.111; p<0.05), mouthguard general knowledge (r=0.364; p<0.01) and mouthguard benefits knowledge (r=0.454; p<0.01) (Table 3).

**Table 3:** Spearman’s Rho correlation analysis between mouthguard usage and demographic parameters

Mouthguard Usage nominal	*r*	*p*
Age	0.065	0.190
Gender	−0.043	0.390
Tooth brush frequency	0.019	0.704
Dentistry visit frequency	0.214^**^	**0.000**
Sport year	0.007	0.882
Sport frequency	0.244^**^	**0.000**
License	0.111^*^	**0.024**
License type	0.101	0.096
Facial dental trauma time	0.000	1.000
Mouthguard general knowledge	0.364^**^	**0.000**
Mouthguard benefits knowledge	0.454^**^	**0.000**

*p < 0.05, **p < 0.01

Binary logistic regression analysis results showed that effects of sport frequency (B = 0.496; *p* < 0.01) and mouthguard benefit knowledge (B = 2.240; *p* < 0.01) on mouthguard usage were statistically significant There was a strong odds ratio (9.396) for mouthguard benefits knowledge (Table 4).

**Table 4:** Binary logistic regression on effects of significantly correlated factors on mouthguard usage

	B	S.E.	Wald	df	*p*	OR	95% C.I.for OR	
							Lower	Upper
Dentistry visit frequency	0.412	0.140	8.643	1	0.003	1.509	1.147	1.986
Sport frequency	0.496	0.177	7.854	1	0.005	1.643	1.161	2.324
License	0.234	0.357	0.430	1	0.512	1.263	0.628	2.541
Mouthguard general knowledge	0.508	0.458	1.229	1	0.268	1.662	0.677	4.083
Mouthguard benefits knowledge	2.240	0.457	23.984	1	0.000	9.396	3.833	23.033
Constant	−4.850	0.568	72.993	1	0.000	0.008		

*B* Regression coefficient, *S.E.* Standard Error, *df* Degree of freedom, *OR* Odds Ratio, *C.I*. Confidence Interval

Among of participants using mouthguards, 25.8% stated that they had used mouthguards for less than a year, 19.7% for 1–2 years, 21.2% for 3–4 years, and 33.3% for 5 years or more. Of those using mouthguards, 15.9% used stock type, 36.2% boiled and bite, 44.9% custom, and 2.9% instrumented type mouthguards. Of the participants, 68.6% stated that their dentists applied mouthguards, 34.3% had airway complications, 40.0% knew the differences between mouthguards, and 67.2% received dental advice when choosing a mouthguard. Usage was generally high in competitions (40.3%) and training (43.3%). Of the participants who used mouthguards, 31.3% stated that they used mouthguards because it was mandatory, 19.4% because of past trauma experience, 14.9% because they had witnessed trauma in the past, and 34.3% for safety. The highest frequency of mouthguard change was once a year with 36.8%, and toothpaste and brush were the most frequently chosen cleaning methods with 44.1%. 62.7% of the participants stated that they regularly cleaned their mouthguards. The most important criteria for selection were comfort and protection with 19.1%. 46.4% of the participants thought that 75% of their teeth would be protected with the mouthguard. The most common difficulty was discomfort with 13.0%. 20.3% of the participants had experienced mouthguard trauma, and 82.6% stated that they purchased mouthguards individually (Table 5).

**Table 5:** Mouthguard usage properties of participants using mouthguard

		Frequency (*n*)	Percent (%)
Mouthguard duration	Under one year	17	25.8
	1–2 years	13	19.7
	3–4 years	14	21.2
	5 years and more	22	33.3
Mouthguard type	Stock Type	11	15.9
	Boil-and-Bite	25	36.2
	Custom	31	44.9
	Instrumented	2	2.9
Customs applied by dentists	No	22	31.4
	Yes	48	68.6
Stocks may cause airway complication	No	46	65.7
	Yes	24	34.3
Difference knowledge between mouthguards	No	42	60.0
	Yes	28	40.0
Dentistry suggestion selecting mouthguard	No	22	32.8
	Yes	45	67.2
Mouthguard time	Training	11	16.4
	Competition	27	40.3
	Both	29	43.3
Mouthguard reason	Compulsory	21	31.3
	Past experienced trauma	13	19.4
	Past seen trauma	10	14.9
	Safety	23	34.3
Mouthguard change frequency	Never	9	13.2
	One in two years	12	17.6
	Once in a year	25	36.8
	Twice in a year	22	32.4
Mouthguard cleaning	With soap and water	8	11.8
	Toothbrush and toothpaste	30	44.1
	Only water	25	36.8
	Detergent etc.	5	7.4
Mouthguard cleaning frequency	Never	1	1.5
	Rarely	24	35.8
	Always	42	62.7
Ideal properties	Cheap	2	2.9
	Durable	2	2.9
	Comfortable	13	19.1
	Protective	13	19.1
	Light	4	5.9
	Remarkable	4	5.9
	Other	30	44.1
How protects	0	2	2.9
	25%	4	5.8
	50%	21	30.4
	75%	32	46.4
	100%	10	14.5
Difficulties	None	16	23.2
	Discomfort	9	13.0
	Breathing problems	7	10.1
	Vomiting	6	8.7
	Bad smell	8	11.6
	Broken	3	4.3
	Allergy	1	1.4
	Other	19	27.5
Mouthguard trauma	No	55	79.7
	Yes	14	20.3
Mouthguard finance	Organization	12	17.4
	Individual	57	82.6

## Discussion

This study examined the knowledge, awareness, and use of mouthguards among amateur and professional athletes. A survey was conducted on 411 athletes. The results revealed that mouthguard use and knowledge were quite low among participants, most of whom participated in sports involving collisions and high risk of oral trauma.

Mouthguards are devices used to protect the soft and hard tissues within the mouth, especially the mouth and eye areas, and teeth in the event of head trauma. They are classified according to their intended use, production method, and purpose [1–5]. Mouthguards are particularly important in dentistry because they facilitate orthodontic treatment following impact and trauma [11]. Literature studies on mouthguard use in dentistry have focused primarily on the interaction between dental health and mouthguard use, knowledge level, usage, and awareness. Mat Zainal et al. [19] reported that the use of custom-fitted mouthguards in rugby athletes did not cause a significant difference in oral function. Padilha et al. [20] also reported that the use of mouthguards in rugby athletes was effective in reducing orofacial trauma, but that custom-made mouthguards, rather than prefabricated mouthguards, should be used. Wang et al. [21] reported that the use of mouthguards not only reduced orofacial trauma in athletes but also improved their athletic performance. Messias et al. [22] reported in their study that the use of custom-made mouthguards provides effective protection for the anatomical structure and roots of teeth, but that more flexible mouthguard designs are needed. Regarding knowledge levels, Haddad et al. [23] reported that using materials such as toothpaste, liquid soap, and water for mouthguard cleaning causes corrosion on surfaces and that awareness levels in this area are low. In our sample, toothpaste and a brush were the most commonly used cleaning products, followed by soap and water. Kasum et al. [24] reported in their study on Croatian soccer players that mouthguard use was quite low among players and knowledge levels were also low. Vignesh et al. [25] reported a generally low level of use and knowledge among sports participants. While the use of mouthguards is particularly effective in sports competitions, their use is limited. A primary reason for the limited use of mouthguards in literature is a lack of knowledge [26–28]. Therefore, more awareness-raising efforts are needed to increase the use of mouthguards, especially in high-intensity sports. For mouthguard benefits knowledge, odds ratio (9.396) was very high to show importance of knowledge. In our study, although the majority participated in sports activities with a high risk of orofacial trauma, mouthguard knowledge and use were limited.

### Limitations of the study

The most significant limitation of the study is its very large population size, as the risk of orofacial trauma spans a very broad spectrum. Therefore, the study population was broad. However, more specific and field-specific studies are needed. Furthermore, the study results can be expanded by multi-center studies.

Another limitation of the study is the lack of adequate measurement tools on this topic. While existing studies consist of demographic questions, measurement tools with established validity and reliability are needed. The limited number of participants in certain sports, such as badminton and athletics, limits the results of the chi-square test. Further studies could explore detailed analyses specific to specific sports.

### The research’s contributions to the literature and clinical practice

The research’s contribution to the literature is that there are few multi-sample studies covering different sports on mouthguard use, and it contributes to addressing this gap. Existing studies are generally limited in terms of either sample size, sport type, or question diversity. In this respect, the research contributes to similar studies in the literature with its sample and findings. Furthermore, no studies were found in the study that analyzed mouthguard use using correlation and regression, or relational screening methods. In this respect, the research contributes methodologically.

The research’s contribution to clinical practice is that it demonstrates the importance and impact of the information provided by dentists on this topic. Therefore, although all stakeholders are important in mouthguard use, multivariate analyses reveal that knowledge about mouthguards increases use, and dentists play a significant role in this regard.

## Conclusion

Although the majority of participants participated in sports involving collisions and those with a high risk of oral trauma, mouthguard use and awareness were quite low. Therefore, dentists, coaches, and public health authorities need to work to encourage mouthguard use.

To ensure more effective use and knowledge in this area, interdisciplinary collaborations such as joint educational programs with the Turkish Football Federation and studies are needed between the field and the field, including public administration. In this regard, research results can be evaluated across disciplines and used as resources for awareness-raising efforts.

- Mouthguard use should be increased for athletes.
- Mouthguard use should be supported for public authorities.
- Mouthguard use should be recommended for coaches.

## Supplementary Information

Supplementary Material 1.

Supplementary Material 2.

## Data Availability

Data of the study is restricted by ethical approval, and may be asked with reasonable manner from corresponding author.
