# Supplementary material for: Mouthguard use and awareness among athletes in Turkey: a cross-sectional study with multivariable analysis
Source: BMC Sports Sci Med Rehabil. 2025 Nov 17;17:339. doi: 10.1186/s13102-025-01377-y (PMC12625523; doi:10.1186/s13102-025-01377-y)
Supplement: Supplementary file 1 — Supplementary Material 1. [file 13102_2025_1377_MOESM1_ESM.docx]

Sportif faaliyetler esnasında ağız koruyucu (mouthguard) kullanımı ve farkındalığı hakkında bir anket çalışması

Değerli Katılımcı;    
Bu anket sportif faaliyetlerde ağız koruyucu (mouthguard) kullanımına yönelik görüşlerinizi değerlendirmek amacıyla yapılmaktadır.
Araştırma bilimsel bir nitelik taşıdığından derlenen kişi ve bilgiler gizli tutulacaktır.
Lütfen soruları tam olarak okuduktan sonra kendinize en uygun olan cevabı işaretleyiniz.
Katkılarınız için şimdiden teşekkür ederiz.

1. Yaşınız?*

2. Cinsiyet ?*

Kadın

Erkek

3. Diş fırçalama sıklığınız?*

Günde 2 kere

Günde 1 kere

Nadiren

Aklıma geldikçe

Hiç

4. Diş hekimine ne sıklıkla başvurursunuz?*

3 ayda bir defa

6 ayda bir defa

Yılda bir defa

Şikayetim olduğu zaman

Bu zamana kadar hiç başvurmadım

5. Hangi spor dalıyla ilgileniyorsunuz?*

Birden fazla seçenek işaretleyebilirsiniz.

Atletizm

Basketbol

Boks

Buz hokeyi

Buz pateni

Dövüş sanatları

Futbol

Hentbol

Kayak

Jimnastik

Masa tenisi

Tenis

Voleybol

Diğer:

6. İlgilendiğiniz sporu kaç yıldır yapıyorsunuz?*

1 yıldan az süredir

1-2 yıl

3-4 yıl

5 veya daha fazla

7. İlgilendiğiniz spor dalını yapma sıklığınız?*

Haftada 1-2 gün

Haftada 3-4 gün

Haftada 5-6 gün

Haftanın her günü

Diğer:

8. Uğraştığınız spor dalında lisansınız var mı?*

Hayır

Evet

9. Cevabınız evet ise; spor lisansınızın türü ne?

Amatör

Profesyonel

10. Fasiyal (yüz) veya dental (dişlerle ilgili) travmaya maruz kaldınız mı?*

Hayır

Evet

11. Cevabınız evet ise antrenman esnasında mı yoksa müsabakada esnasında mı?

Antrenman esnasında

Müsabaka esnasında

12. Ağız koruyucu (Mouthguard) ile ilgili bilginiz var mı?*

Hayır

Evet

13. Mouthguardın faydalarını biliyor musunuz?*

Hayır

Evet

14. Mouthguard kullanıyor musunuz?*

Hayır, kullanmıyorum

Evet, düzenli kullanıyorum

Bazen kullanıyorum

15. Cevabınız evet ise mouthguardı ne zamandır kullanıyosunuz?

1 yıldan az

1-2 yıl

3-4 yıl

5 yıl ve daha fazlası

Travma sonrası kullanmaya başladım

16. Hangi tür mouthguard kullanıyorsunuz?

Stock Tip (Hazır prefabrik)

Boil-and-Bite (Kaynat ve ısır)

Custom (Kişiye özel)

Instrumented (Teknolojik)

17. Kişiye özel mouthguard'ların diş hekimleri tarafından yapıldığını biliyor musunuz?*

Hayır

Evet

18. Stok tip ağız koruyucu (mouthguard) kullanımının hava yolu tıkanması dahil komplikasyonlara sebep olabileceğini biliyor musunuz?*

Hayır

Evet

19. Stok, boil and bite ve custom ağız koruyucularının farklılıklarıyla ilgili bilginiz var mı?*

Hayır

Evet

20.  Mouthguard seçimi yaparken diş hekiminden yardım alıyor musunuz?

Hayır

Evet

21. Mouthguardı hangi aşamada kullanıyosunuz?

Antreman

Müsabaka

Antreman ve müsabaka

22. Mouthguard kullanımını neden tercih etmektesiniz?

Zorunlu tutulduğu için

Daha önce travma yaşadığım için

Daha önce travmaya şahit olduğum için

Güven hissettirdiği için

23. Mouthguard'larınızı ne sıklıkta değiştiriyorsunuz?

6 ayda bir

Yılda bir

2 yılda bir

Hiç değiştirmedim

24. Mouthguard temizliğinizi nasıl yapıyorsunuz?

Sabun ve su ile

Diş fırçası ve macun yardımıyla

Sadece su ile

Deterjan vb. ile

Diğer:

25. Mouthguard temizliğini ne sıklıkta yapıyorsunuz?

Her kullandığımda

Bazen

Hiç temizlemiyorum

26. İdeal bir mouthguard'ta aradığınız özellikler ve beklentileriniz nedir?

Birden fazla seçenek işaretleyebilirsiniz.

Hafif olması

Dikkat çekici olması

Koruyucu olması

Rahat olması

Ucuz olması

Sağlam olması

27. Mouthguard'ın sizi ne kadar koruduğuna inanmaktasınız?

%100

%75

%50

%25

%0

28. Mouthguard kullanırken aşağıdaki zorluklardan hangisi veya hangilerini yaşadınız?

Birden fazla seçenek işaretleyebilirsiniz.

Kırılma

Kusma

Kötü koku

Nefes alma güçlüğü

Rahatsızlık hissi

Alerji

Hiçbir zorluk yaşamadım

29. Mouthguard kullanırken travma yaşadığınız mı?

Hayır

Evet

30. Mouthguard'ınızı kurum mu karşılıyor bireysel mi karşılıyorsunuz?

Kurum

Bireysel
