# Supplementary material for: Mouthguard use and awareness among athletes in Turkey: a cross-sectional study with multivariable analysis
Source: BMC Sports Sci Med Rehabil. 2025 Nov 17;17:339. doi: 10.1186/s13102-025-01377-y (PMC12625523; doi:10.1186/s13102-025-01377-y)
Supplement: Supplementary file 2 — Supplementary Material 2. [file 13102_2025_1377_MOESM2_ESM.docx]

A survey study on mouthguard use and awareness during sporting activities

Dear Participant,
This survey is being conducted to assess your opinions regarding the use of mouthguards in sporting activities. Because the research is scientific in nature, the individuals and information collected will be kept confidential. Please read the questions thoroughly and select the answer that best suits you. Thank you in advance for your contributions.

1. What is your age? *

2. Gender? *

Female

Male

3. How often do you brush your teeth? *

Twice a day

Once a day

Rarely

As I remember

None

4. How often do you visit the dentist? *

Once every 3 months

Once every 6 months

Once a year

When I have a complaint

I have never applied until now

5. What kind of sport are you interested in? *

You can select more than one option.

Athletics

Basketball

Boxing

Ice hockey

Ice skating

Martial arts

Football

Handball

Ski

Gymnastics

Table tennis

Tennis

Volleyball

Other:

6. How many years have you been doing the sport you are interested in? *

Less than 1 year

1-2 years

3-4 years

5 or more

7. How often do you do the sport you are interested in? *

1-2 days a week

3-4 days a week

5-6 days a week

Every day of the week

Other:

8. Do you have a license in the sport you practice? *

No

Yes

9. If your answer is yes, what type of sports license do you have?

Amateur

Professional

10. Have you ever suffered facial or dental trauma? *

No

Yes

11. If yes, during training or competition?

During training

During the competition

12. Do you have any information about mouthguards? *

No

Yes

13. Do you know the benefits of mouthguard? *

No

Yes

14. Do you use mouthguard? *

No, I don't use it

Yes, I use it regularly

I use it sometimes

15. If your answer is yes, how long have you been using the mouthguard?

Less than 1 year

1-2 years

3-4 years

5 years and more

I started using it after a trauma

16. What type of mouthguard do you use?

Stock Type (Ready-made prefabricated)

Boil-and-Bite

Custom (Personalized)

Instrumented (Technological)

17. Did you know that custom mouthguards are made by dentists? *

No

Yes

18. Did you know that the use of a stock-type mouthguard can cause complications, including airway obstruction? *

No

Yes

19. Do you have any information about the differences between stock, boil and bite, and custom mouthguards? *

No

Yes

20. Do you get help from a dentist when choosing a mouthguard?

No

Yes

21. At what stage do you use mouthguard?

Workout

Competition

Training and competition

22. Why do you prefer using mouthguard?

Because it is mandatory

Because I've been traumatized before

Because I have witnessed trauma before

Because it makes you feel safe

23. How often do you change your mouthguards?

Every 6 months

Once a year

Every 2 years

I never changed it

24. How do you clean your mouthguard?

With soap and water

With the help of a toothbrush and paste

Only with water

With detergent etc.

Other:

25. How often do you clean your mouthguard?

Every time I use it

Sometimes

I never clean it

26. What are the features and expectations you look for in an ideal mouthguard?

You can select more than one option.

Being light

Being eye-catching

Being protective

Being comfortable

Being cheap

Being solid

27. How well do you believe Mouthguard protects you?

100%

75%

50%

25%

0%

28. Which of the following difficulties did you experience while using mouthguard?

You can select more than one option.

Breaking

Vomiting

bad smell

Difficulty breathing

Feeling of discomfort

Allergy

I had no difficulties

29. Have you experienced trauma while using mouthguard?

No

Yes

30. Do you pay for your mouthguard through an institution or individually?

Organisation

Individual
